# Preliminary Computational Hemodynamics Study of Double Aortic Aneurysms under Multistage Surgical Procedures: An Idealised Model Study

**DOI:** 10.1155/2013/601470

**Published:** 2013-11-17

**Authors:** Yosuke Otsuki, Nhat Bui Minh, Hiroshi Ohtake, Go Watanabe, Teruo Matsuzawa

**Affiliations:** ^1^School of Information Science, Japan Advanced Institute of Science and Technology, 1-1 Asahidai, Nomi, Ishikawa 923-1211, Japan; ^2^Department of Simulation Analysis, Fujitsu Systems East, 1415 Tsuruga Midori, Nagano 380-0813, Japan; ^3^Department of General and Cardiothoracic Surgery, Graduate School of Medical Science, 13-1 Takara, Kanazawa 920-1192, Japan; ^4^Research Center for Simulation Science, Japan Advanced Institute of Science and Technology, 1-1 Asahidai, Nomi, Ishikawa 923-1211, Japan

## Abstract

Double aortic aneurysm (DAA) falls under the category of multiple aortic aneurysms. Repair is generally done through staged surgery due to low invasiveness. In this approach, one aneurysm is cured per operation. Therefore, two operations are required for DAA. However, post-first-surgery rupture cases have been reported. Although the problems involved with managing staged surgery have been discussed for more than 30 years, investigation from a hemodynamic perspective has not been attempted. Hence, this is the first computational fluid dynamics approach to the DAA problem. Three idealized geometries were prepared: presurgery, thoracic aortic aneurysm (TAA) cured, and abdominal aortic aneurysm (AAA) cured. By applying identical boundary conditions for flow rate and pressure, the Navier-Stokes equation and continuity equations were solved under the Newtonian fluid assumption. Average pressure in TAA was increased by AAA repair. On the other hand, average pressure in AAA was decreased after TAA repair. Average wall shear stress was decreased at the peak in post-first-surgery models. However, the wave profile of TAA average wall shear stress was changed in the late systole phase after AAA repair. Since the average wall shear stress in the post-first-surgery models decreased and pressure at TAA after AAA repair increased, the TAA might be treated first to prevent rupture.

## 1. Introduction

An aortic aneurysm is abnormal dilatation on the aortic wall. The formation and growth process are not fully understood although some fundamental biological evidence has been identified [1]. Lasheras stated that repeatedly applied shear force stiffens the aortic wall by changing the biomechanical reaction and when the aneurysm wall strength can no longer tolerate the mechanical load, it ruptures. Moreover, he also mentioned that atherosclerotic plaque was responsible for the growth since it was found in the majority of aneurysms. The death rate due to rupture is high; according to Shek, it reaches 85% if an abdominal aortic aneurysm (AAA) is left untreated [2]. The size of an aneurysm is closely related to the rupture risk. Choke et al. demonstrated the relationship between the size of AAA and the annual risk of rupture [3]. From their research, the rupture risk increased as the aneurysm diameter increased. Population-based research shows that aneurysms are frequently formed at the aortic arch and abdominal wall [4].

To cure an aneurysm, stent-graft implantation or artificial graft replacement is the common surgical procedure. Since the first procedure is less invasive, it is frequently used. Frauenfelder et al. illustrated the reduction in AAA pressure by stent-graft implantation through their fluid structure interaction model and validation experiment [5].

If more than one aortic aneurysm is found, the condition is called multiple aortic aneurysms (MAA). Crawford and Cohen reported that 191 of 1510 (12.6%) aneurysm patients were diagnosed as MAA [4]. Yamanaka et al. also reported that 153 of 1750 (8.74%) aneurysm patients had MAA [6]. Two surgical approaches are used for treating MAA: staged surgical treatment or simultaneous surgery. The former repairs each aneurysm by a single operation after a certain recovery period; the latter treats all aneurysms by a single operation. Crawford and Cohen suggested that simultaneous surgery should be used to prevent death due to rupture of the second aneurysm during the recovery period [4]. However, the staged method is generally used because of its high surviving rate [7]. The problem with the staged approach is the management difficulty between operations: rupture of residual aneurysms during this period has been reported. Yamazaki et al. reported TAA rupture after successful AAA stent graft implantation [8] and Ohnishi et al. also reported similar cases [9]. Crawford and Cohen explained that postoperation early death is caused by the second aneurysm rupturing [4]. Normally, surgery priority is determined based on the size of aneurysm and other surgical indications. The indication size is five centimeters in diameter for AAA and six centimeters for TAA. This is a reasonable approach since the size of aneurysm is closely related to the rupture risk. However, Kanamitsu and Yamada explained that AAA repair is generally prioritized since the surgery of TAA alone is high risk [10].

MAA-specific problems can be categorized as follows. First, it is not clear whether TAA or AAA repair should be prioritized. Second, the timing of surgery is determined solely by the size of aneurysm. To clarify these points, computational fluid dynamics was used.

Thanks to the development of diagnostic equipment, information taken from patients can be analyzed before surgery. Blood flow velocity can be measured using magnetic resonance imaging or ultrasound devices. However, crucial information such as pressure and wall shear stress cannot be sampled by these methods. The computational approach enables these variables to be obtained, and *in vivo* conditions can be simulated. From the perspective of hemodynamics, a number of experimental and computational studies for single aneurysms have been conducted. Since aneurysms are found in thoracic and abdominal parts, previous works focused on TAA or AAA [5, 11]. However, there are no reports on MAA.

Hence, the present research had two objectives. The first was to investigate the change in flow pattern, wall shear stress, and pressure distribution after the treatment of AAA and TAA, respectively. The second was to identify, based on these results, the lowest risk-staged surgery for treatment of double aortic aneurysms (DAA). DAA, which has two aneurysms, is the simplest type of MAA. To clarify these factors, three idealized aorta models were prepared.

The models represented the untreated condition before surgery (DAA model), TAA removed condition after the first surgery (AAA cured model), and AAA repaired geometry after the first surgery (TAA cured model). Next, flow was simulated using two pairs of boundary conditions. Then, the results from the postsurgery geometries were compared with the result from the presurgery model.

## 2. Methods

As discussed, changes in wall shear stress and pressure distribution are considered to be important factors for aneurysms. Therefore, two cases of boundary conditions were used in the present study. The first case was the fixed flow rate boundary condition. Since wall shear stress is a function of only velocity and coordinates, if the inlet flow rate is fixed, a change in wall shear stress is attributed to the geometrical difference under incompressible and the fluid assumption. The second case was the pressure difference boundary condition.

In this method, to investigate the effect of time-dependent pressure inlet and outlet pressure profiles were used. In both cases, calculation was repeated five times.

### 2.1. Geometry Construction

**Figure 1:**
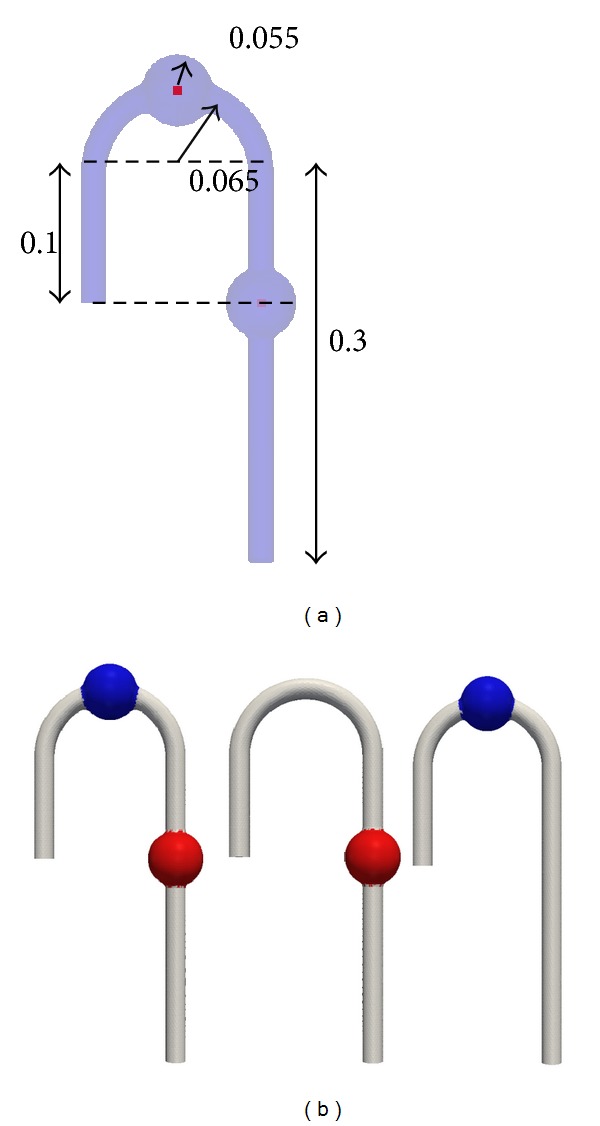
DAA (left), TAA cured (centre), and AAA cured (right) geometries, red: abdominal aortic aneurysm, blue: thoracic aortic aneurysm. Inlet: left, outlet: right, all dimensions in meter.

The model geometries are shown in Figure 1. For the TAA and AAA cured models, the repaired model aneurysm section was straight, assuming stent-graft insertion. The geometries and computation mesh were created using Gambit 2.3.6 (ANSYS Inc.). The aneurysm diameter of 0.055 m (5.5 cm) was determined by Greenhalgh et al. [12] as the surgery criterion for AAA. The aorta diameter of 0.02 m (2 cm) and straight pipe length were determined by Gao et al. [13]. However, an extra length of 0.1 m (10 cm) was added to the inlet. The arch diameter of 0.13 m (13 cm) was determined by the double aortic aneurysm geometry reconstructed from computed tomography data. At the intersection edge of the straight model artery and the aneurysm, a fillet of 0.005 m (0.5 cm) was applied. All aortic branches were ignored. The locations of the aneurysms were determined in the aortic arch and abdominal part according to Crawford and Cohen [4]. A tetrahedron mesh was created using Gambit. The mesh characteristic size was set to 0.001 m (0.1 cm). The number of elements was 80103, 71748, and 69342 for the DAA, TAA cured, and AAA cured model, respectively.

### 2.2. Computational Fluid Dynamics (CFD)

For the simulation, the commercial CFD package Fluent 14.5 (ANSYS Inc.) was used. The Navier-Stokes and continuity equations were discretized by the finite volume method assuming Newtonian laminar flow and were solved. For density and viscosity, 1050 kg m^−3^ and 0.0035 N m^−1^ s^−1^ were assigned.

### 2.3. Boundary Condition

As explained earlier, the fixed flow rate and fixed pressure boundary conditions were simulated to observe changes in wall shear stress and pressure after the first surgery. 

**Figure 2:**
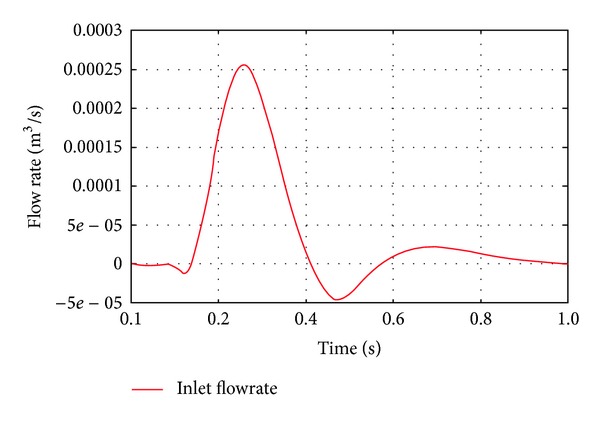
Inlet flow rate profile for flow rate fixed simulation.

**Figure 3:**
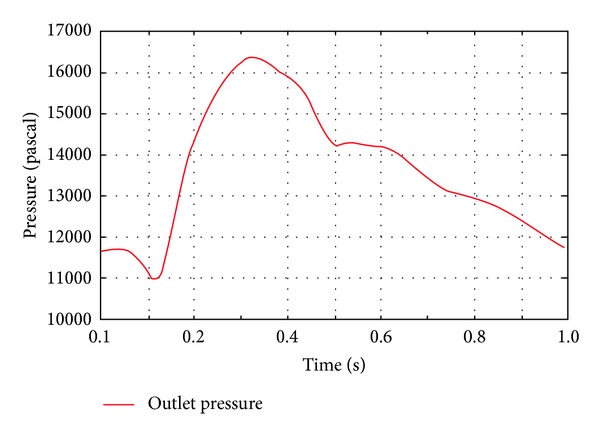
Outlet pressure profile.

**Figure 4:**
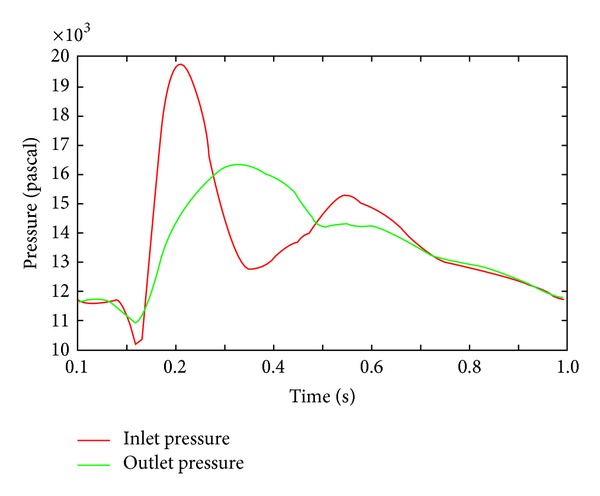
Calculated inlet pressure profile (red) and outlet profile (green) for pressure fixed simulation.

Therefore, two pairs of boundary conditions were prepared. First, the inflow rate profile was obtained from Tse et al. [14], and the pressure profile from Olfusen et al. [15] was used for wall shear stress analysis. The pressure curve at the inlet was sampled and then was used as the boundary condition for the fixed pressure calculation. The pressure was sampled at the centre of the inlet. The spatial variation on the inlet cross-section was negligible. The flow rate and pressure curve are shown in Figures 2 and 3, respectively. The calculated pressure wave profile at the inlet and the outlet pressure profile are shown in Figure 4. To sample inlet pressure curve, the DAA model was used. In Figure 4, the red line indicates the calculated pressure and the green line represents the outlet pressure. These curves were uniformly applied at the inlet and outlet planes.

## 3. Result

The velocity vector and wall shear stress from the fixed flow rate model boundary condition and the pressure sampled from the fixed pressure difference boundary condition are shown. For the flow pattern analysis, the results from the fixed flow rate and fixed pressure boundary conditions will be presented. For the analysis of wall shear stress, the results were taken from the fixed flow rate case, and for the analysis of pressure, the pressure difference boundary condition results were used. The average Reynolds number was 925 and the maximum Reynolds number was 4880 according to the flow rate profile and the geometry. The calculation result was taken from the fifth cycle (*t* = 4.0-5.0 s).

### 3.1. Velocity

**Figure 5:**
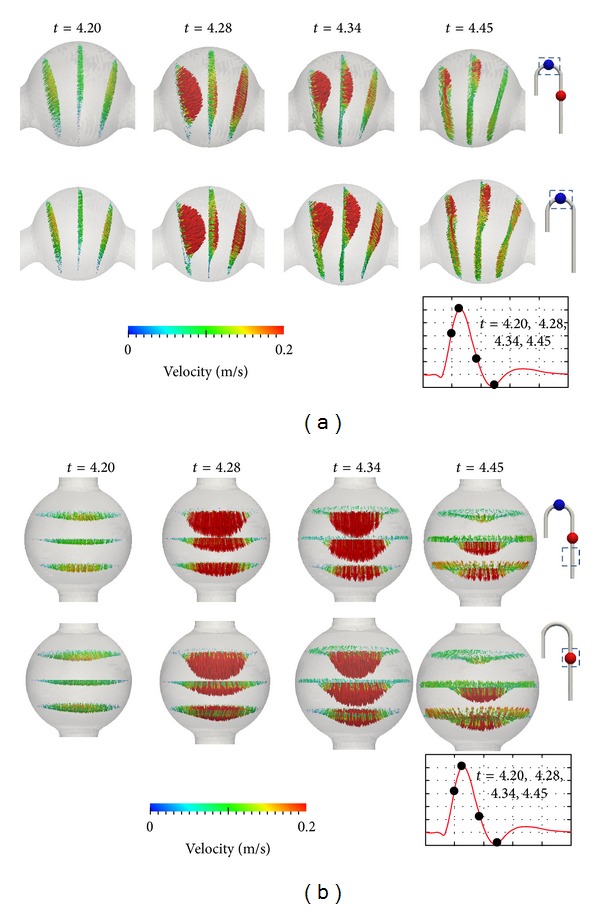
Velocity vector in aneurysms: (a) TAA in DAA model versus TAA in AAA cured model, (b) AAA in DAA model versus TAA cured model.

In the early and late systole phases, vortices were created in TAA and AAA. When the flow accelerated, vortices disappeared. The velocity magnitude was smaller in an aneurysm than in the straight artery section.  Figure 5 plots the velocity vector in aneurysms. The figure uses the fixed flow rate boundary condition to demonstrate the relationship with the distribution of wall shear stress. The colour of the vector illustrates the magnitude of velocity. The images were taken at *t* = 4.20, 4.28, 3.34, and 4.45 s. These represent early systole, peak, late systole, and backflow phases. For the TAA flow pattern in the DAA and AAA cured models, there was no vortex during the acceleration. In the early and the late systole phase, vortices were created in TAA and AAA. When the flow accelerated, vortices disappeared. In aneurysm, the velocity magnitude was slower than the straight tube section. As shown in Figure 5(a), there is no backflow or vortex at *t* = 4.28 s. As the velocity decreased, a clockwise vortex was formed in TAA. At this time step, the flow entered the aneurysm from the outer aortic arch (*t* = 4.34 s in Figure 5(a)). A small backflow was initiated on the inner aortic arch wall, and the backflow velocity magnitude gradually increased after the peak. As shown in Figure 5(a), the circulation velocity magnitude at this time step was larger in the AAA cured model than in the DAA model. For AAA flow in the DAA and TAA cured models, almost symmetrical vortices were found for most time steps.  Figure 5 shows that, at *t* = 4.34 s, flow faster than 0.2 m s^−1^ was more concentrated at the central path in the DAA model than in the TAA cured model. At *t* = 4.34 and 4.45 s, backflow near the wall can be observed in both the AAA in DAA and in TAA cured models. However, the symmetry was temporarily destroyed at the initial backflow, which can be confirmed from the velocity vector plot at *t* = 4.45 s in the TAA cured model.

**Figure 6:**
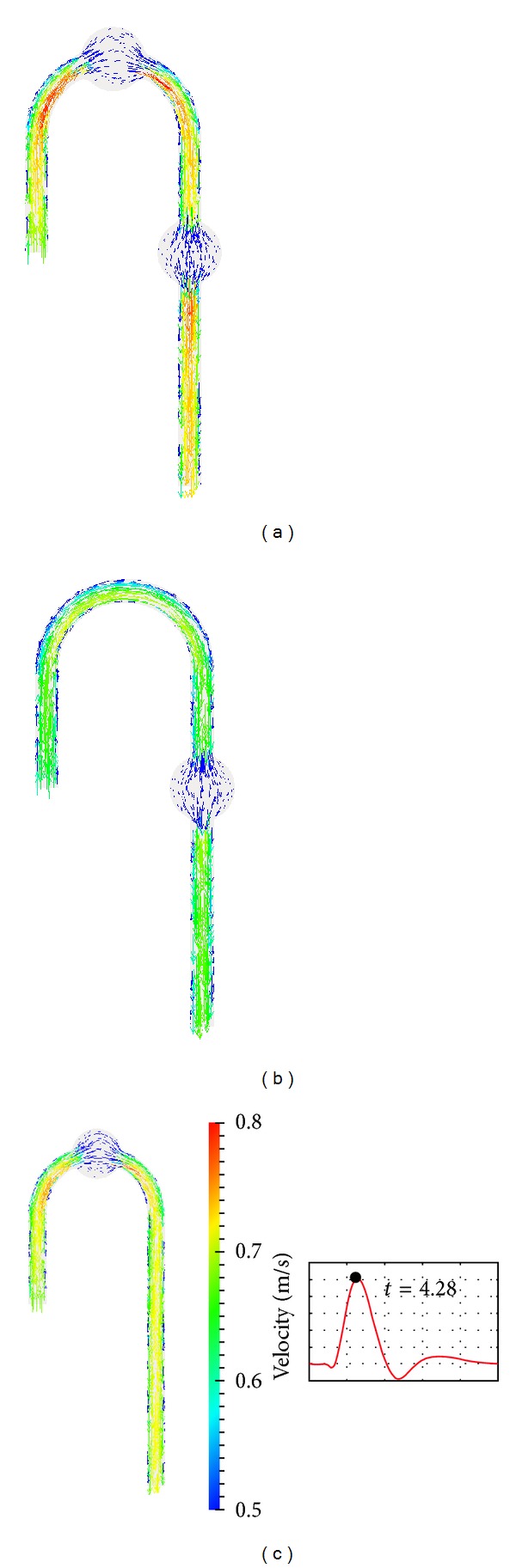
Peak velocity on DAA, TAA cured, and AAA cured middle plane DAA (a), TAA cured (b), and AAA cured (c).

**Figure 7:**
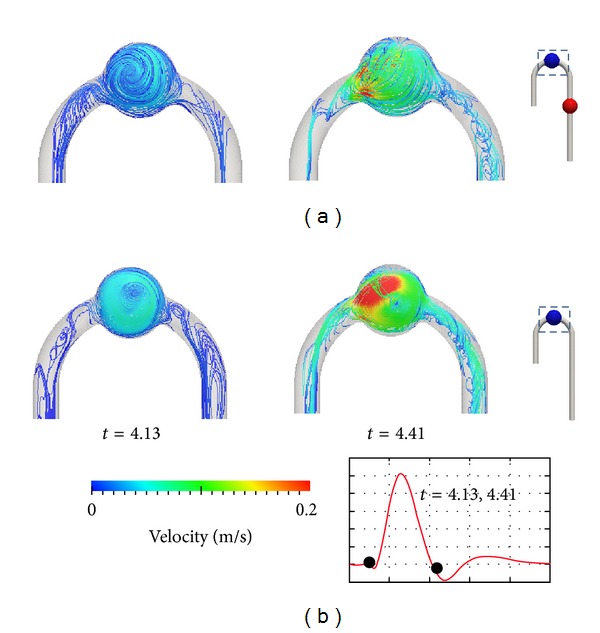
Helical flow at ascending and descending aorta *t* = 4.13 and *t* = 4.41 second: (a) DAA, (b) TAA cured (right).

To observe the geometrical effects on the flow, the velocity vector plot on the middle planes of the model is illustrated in Figure 6. The figure was taken at the velocity peak *t* = 4.28 s. The colour illustrates the magnitude of velocity. The flow velocity magnitude in the cured models was clearly slower than in the DAA model. The slowest flow speed was found in the AAA cured model. The TAA cured model had the second largest velocity magnitude at the peak. However, at the mid-to-late systole phase, the velocity magnitude in the TAA cured and AAA cured models increased, and a larger increase was found in the TAA model. Fast flow was found at the inner wall of the aortic arch for all models. The velocity magnitude of the AAA cured model was the smallest in this section.  Figure 7 depicts helical flow by streamline plots; color illustrates the magnitude of velocity. At both *t* = 4.13 and 4.41 s, fast circulation flow was found in TAA. At *t* = 4.41 s, there was a difference of over 0.2 m s^−1^ in the region near the inlet. Helical flow was observed at the ascending and descending aorta, which is a known characteristic for the aortic arch flow [16].

Furthermore, the flow was observed several time steps before the acceleration of velocity.

### 3.2. Pressure

**Figure 8:**
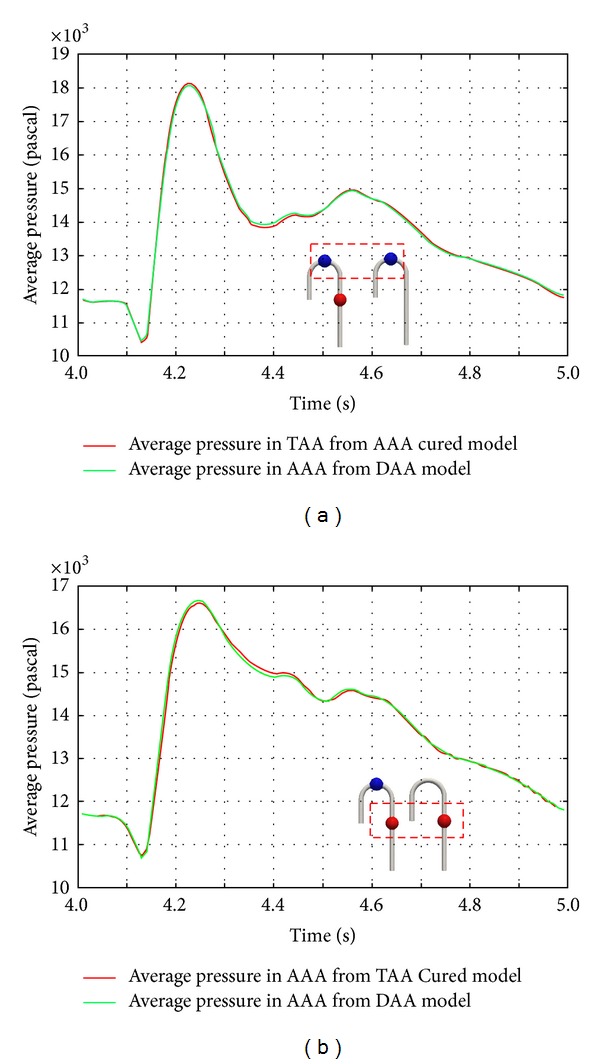
Pre- and post-first-surgery average pressure change in aneurysms: (a) average pressure comparison in TAA, (b) average pressure comparison in AAA.

Average pressure was calculated to evaluate rupture risk. Pressure was sampled from the colored area in Figure 1. The number of sampling points was 4468 and 4548 for TAA and AAA in the DAA model, respectively. For the AAA cured and TAA cured models, the number was 5060 and 4818 points, respectively. The aneurysm sections had identical surface area. However, because of the difference in sampling node number averaging was required. The change in average pressure in the aneurysm sections is illustrated in Figure 8. In the figure, the red line represents average pressure from DAA mode and the green line illustrates that from AAA model (a)/TAA model (b). A comparison of pre- and postsurgery TAA pressure in Figure 8 indicates that the average pressure increases at *t* = 4.23 s in the AAA cured geometry. Moreover, at *t* = 4.38 s, the TAA pressure in the AAA cured model decreased. Therefore, the magnitude of the pressure wave increased after AAA repair. On the other hand, in the TAA cured model, the AAA pressure decreased at *t* = 4.25 s. Another notable change was between *t* = 4.3 and 4.4 s, when the average pressure in the AAA cured model was smaller than that in the DAA model. The quantitative difference after repair was as follows. For TAA, the increase in pressure was measured to be about 0.8% at the average pressure peak. For AAA, the decrease in pressure was about 0.8% at the average pressure peak. However, the largest pressure change was found at the slope just after the average pressure peak. A decrease of about 1.0% was found from TAA in the AAA cured model, and the figure from AAA in the TAA cured model showed an increase of 1.0%.

### 3.3. Wall Shear Distribution

**Figure 9:**
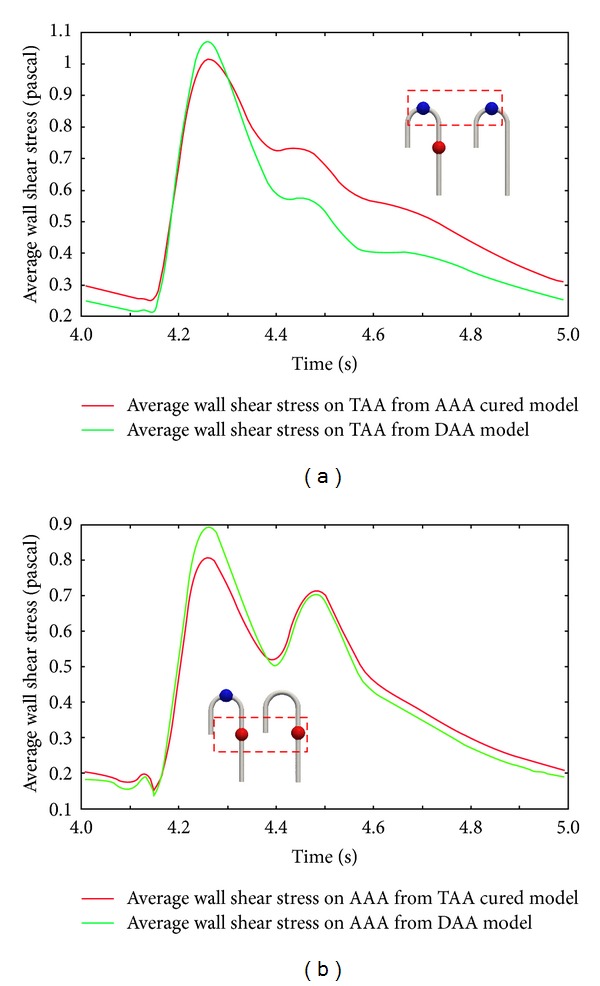
Average wall shear stress per cycle: (a) average wall shear stress comparison in TAA, (b) average wall shear stress comparison in AAA green line. Presurgery double aneurysms, red line: post surgery TAA cured or AAA cured.

Wall shear distribution contours were taken from the fixed flow rate simulation.  Figure 9 shows the average wall shear stress on the TAA surface. The green line represents the average wall shear stress taken from the DAA model, and the red line demonstrates that from the AAA cured model. The sampling sections are coloured in Figure 1. From Figure 9, a comparison between the DAA and AAA cured models shows a drop in average wall shear stress in the AAA cured model at the peak of *t* = 4.28 s. In contrast, average wall shear stress increased after the peak. Similarly, regarding AAA in the DAA and TAA cured models, the average wall shear stress decreased at the peak but increased in the mid-to-late systole phases. By comparing the average wall shear stress taken from TAA and AAA, a larger increase in wall shear stress was found in TAA in the AAA cured model.

**Figure 10:**
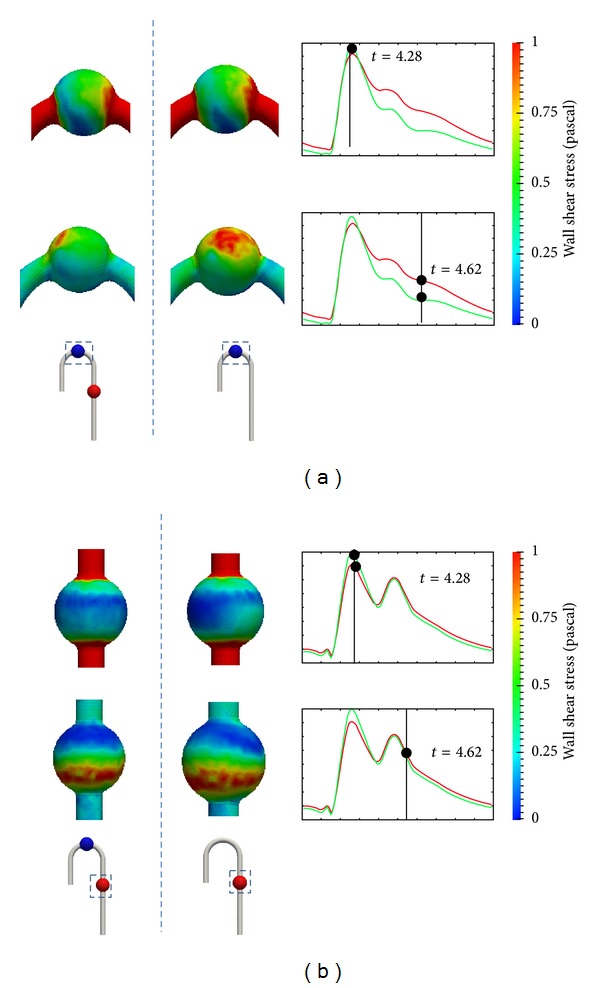
Wall shear stress contour comparison: (a) TAA, (b) AAA.

**Figure 11:**
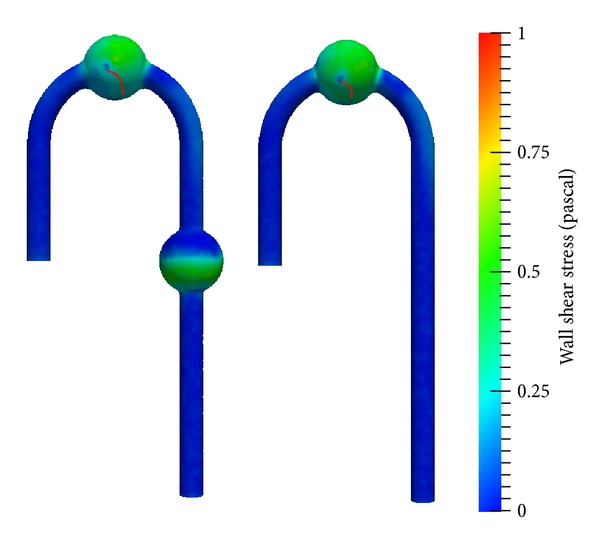
Low wall shear stress point movement path (red line), left: DAA, right: AAA cured.

Furthermore, the late systole wave profile implies that the wall shear stress wave profile differed from that of DAA.  Figure 10 compares the wall shear stress contours at *t* = 4.28 and *t* = 4.62 s. The colour in the figure represents the magnitude of wall shear stress. A comparison of the distribution of TAA wall shear stress from the DAA and AAA cured models reveals a notable difference in the wall shear stress pattern. From TAA in the AAA cured model, a region of wall shear stress of over 1.0 Pa was found in the late systole phase at *t* = 4.62 s. The location was on the wall surface of the outer aortic arch. The wall shear stress of the inner aortic arch was similar in magnitude to that from DAA. The wall shear stress region from the DAA model was around 0.5 Pa. In addition, from the DAA and AAA cured models, a low stress point was found near the TAA center. This location was identical to the center of the vortex. The point movement path is illustrated in Figure 11.

In the AAA wall shear stress distribution, a change in wall shear stress distribution of the posterior side was found although the difference between the DAA and TAA cured models was small. A comparison of AAA in the DAA and TAA cured models reveals that the symmetry of wall shear distribution was lost after TAA repair. At most time steps, the wall shear stress of the posterior side was not equivalent to that of the anterior side in the TAA cured model. At the late systole phase, both the DAA and TAA cured wall shear distributions show a low wall shear stress area from the inlet to the middle of the aneurysm. The region moved downstream during the midsystole phase.

## 4. Discussion

Figure 6 shows that the velocity magnitude in the aneurysm cured models was slower than that in the DAA model at the peak. This is a reasonable result since shear stress is a function of the first derivative of velocity with respect to the distance from the wall, and the viscosity was assumed to be constant in this simulation. If an aneurysm that has a larger diameter than the mother vessel is removed, the distance to the wall must be reduced. In other words, the removal of the aneurysm increases the resistive force. The increase in velocity magnitude in the mid-to-late systole phase was considered to be the cause of the increase in wall shear stress.  Figure 5 at *t* = 4.45 s demonstrates the loss of symmetry after TAA repair. It is considered that the vortex in TAA acted as a mixer or diffuser. In AAA in TAA cured model, the convective effect became larger and the flow symmetry was destroyed.

A comparison of the average aneurysm pressure revealed an increase in pressure at TAA in the AAA cured model. Moreover, the magnitude of the TAA pressure wave increased over the cycle. The authors believe that this may lead to postsurgery TAA failure since an aneurysm rupture is considered to be due to mechanical failure If this assumption is correct, an increase in pressure peak has the largest possibility of rupture. 

A comparison of average wall shear stress in TAA and AAA showed a decrease in the peak and an increase in the late systole phase. A notable change was found at TAA in the AAA cured model, and the increase in average wall shear stress was larger than that at AAA in the TAA cured model. An increase in velocity magnitude after the velocity peak in the TAA and AAA cured models was also confirmed. Moreover, the mid-to-late systole average wall shear stress wave profile changed at TAA in the AAA cured model. From a biomechanics perspective, it is known that the receptor reaction is triggered by a change in wall shear stress distribution and causes solidification of the vessel. Therefore, TAA in the AAA cured model is considered to be a larger risk than AAA in the TAA cured model. A comparison between Figures 5 and 10 demonstrates that the high wall shear stress region matches the region of larger velocity magnitude. For example, when the flow decelerated, the region of larger velocity magnitude moved to the outer aortic arch wall. Regarding the spatial gradient of wall shear stress in TAA, the wall shear stress of the inner aortic arch was smaller than that of the outer aortic arch for all time steps. 

Furthermore, from Figure 11, the low wall shear stress area was found in the center of TAA from the DAA and AAA cured models. The point moved and joined the inner small wall shear stress region just before the acceleration of flow. Since the small shear stress region is closely linked with the accumulation of thrombus [17], this process may work negatively.

Thrombus collected at the center of TAA and moved toward the inner arch wall area and accumulation started there. In addition, Boussel et al. reported the correlation between small wall shear stress area and aneurysm growth [18]. Therefore, for the long term, attention should be paid to the section. In AAA, the wall shear stress near the inlet was smaller than that near the outlet, and the anterior side wall had slightly larger wall shear stress than the posterior side wall. The difference between wall shear stress in the anterior and posterior walls was smaller than that in the inlet and outlet walls. As discussed, the pressure and wall shear stress results suggest an increase in rupture risk in TAA after treatment of AAA, although it is difficult to predict and specify which of the two will trigger this.

There are some limitations in this work. The distribution of inlet velocity was assumed to be uniform, but it is asymmetric in reality. Renner et al. [19] pointed out this problem through simulation from three boundary configurations. In their work, MRI-captured actual velocity distribution, uniform distribution, and parabolic distribution were used to investigate the change in wall shear stress distribution.

To simplify the simulation and to clearly observe the difference in poststent implanted condition, the geometries were assumed to be rigid. If an elastic model is used, a wave shift might be found by fluid and structure coupling effects.

Furthermore, the sizes of TAA and AAA were assumed to be identical to simplify the problem. However, the volume, shape, and location of the geometries differ for each patient. Moreover, the poststent implanted condition was modelled by straight tube. In reality, the surface of the artery is covered by fabric and metal mesh. Therefore, disturbance of the flow cannot be eradicated as demonstrated in this model study.

## 5. Conclusion

To investigate the postsurgery rupture of double aortic aneurysms, changes in pressure and wall shear distribution were investigated under fixed flow rate and pressure difference boundary conditions. The average pressure sampling results revealed that pressure increased at TAA after AAA repair. In contrast, AAA pressure dropped in the TAA cured model. The average wall shear stress demonstrated a decrease at the peak. However, an increase in wall shear stress was found in the late systole phase. In addition, a larger increase was found at TAA in the AAA cured model than that at AAA in the TAA cured model. The calculated average wall shear stress curve differed from the curve for TAA in the DAA model. Since aneurysm rupture is considered to be a mechanical failure, it is reasonable to estimate that rupture is triggered when the maximum load is applied to the structure. Therefore, an increase in average pressure may be one cause of rupture.

Moreover, the increase and change in average wall shear stress curve for TAA in the AAA cured model are also considered to be a cause of rupture in the long term since a change in wall shear stress waveform triggers a biomechanical reaction and solidifies the artery. A less flexible artery is easily damaged. Hence, both the calculated pressure and wall shear stress suggest that the risk of TAA rupture is increased after AAA treatment. Moreover, since AAA pressure decreased and there was a significant difference in the average wall shear stress after TAA repair, TAA treatment should be prioritised.
